# Clinical Outcomes and Prognostic Factors In Triple-Negative Invasive Lobular Carcinoma of the Breast

**DOI:** 10.21203/rs.3.rs-2658909/v1

**Published:** 2023-03-20

**Authors:** Utsav Joshi, Pravash Budhathoki, Suman Gaire, Sumeet K. Yadav, Anish Shah, Anurag Adhikari, Grace Choong, Rima Couzi, Karthik Giridhar, Roberto Leon-Ferre, Judy C. Boughey, Tina J. Hieken, Robert Mutter, Kathryn J. Ruddy, Tufia C. Haddad, Matthew P. Goetz, Fergus J. Couch, Siddhartha Yadav

**Affiliations:** Rochester General Hospital; BronxCare Health System; Mount Sinai Hospital; Mayo Clinic Health System; BronxCare Health System; Jacobi Medical Center; Mayo Clinic Rochester; Johns Hopkins School of Medicine Department of Oncology: Johns Hopkins Medicine Sidney Kimmel Comprehensive Cancer Center; Mayo Clinic Rochester; Mayo Clinic Rochester; Mayo Clinic Rochester; Mayo Clinic Rochester; Mayo Clinic Rochester; Mayo Clinic Rochester; Mayo Clinic Rochester; Mayo Clinic Rochester; Mayo Clinic Rochester; Mayo Clinic Rochester

**Keywords:** Triple negative, lobular breast cancer, neoadjuvant, survival, chemotherapy

## Abstract

**Purpose:**

Triple-negative invasive lobular carcinoma (TN-ILC) of breast cancer is a rare disease and the clinical outcomes and prognostic factors are not well-defined.

**Methods:**

Women with stage I-III TN-ILC or triple-negative invasive ductal carcinoma (TN-IDC) of the breast undergoing mastectomy or breast-conserving surgery between 2010 and 2018 in the National Cancer Database were included. Kaplan-Meier curves and multivariate Cox proportional hazard regression were used to compare overall survival (OS) and evaluate prognostic factors. Multivariate logistic regression was performed to analyze the factors associated with pathological response to neoadjuvant chemotherapy.

**Results:**

The median age at diagnosis for women with TN-ILC was 67 years compared to 58 years in TN-IDC (p<0.001). There was no significant difference in the OS between TN-ILC and TN-IDC in multivariate analysis (HR 0.96, p=0.44). Black race and higher TNM stage were associated with worse OS, whereas receipt of chemotherapy or radiation was associated with better OS in TN-ILC. Among women with TN-ILC receiving neoadjuvant chemotherapy, the 5-year OS was 77.3% in women with a complete pathological response (pCR) compared to 39.8% in women without any response. The odds of achieving pCR following neoadjuvant chemotherapy were significantly lower in women with TN-ILC compared to TN-IDC (OR 0.53, p<0.001).

**Conclusion:**

Women with TN-ILC are older at diagnosis but have similar OS compared to TN-IDC after adjusting for tumor and demographic characteristics. Administration of chemotherapy was associated with improved OS in TN-ILC, but women with TN-ILC were less likely to achieve complete response to neoadjuvant therapy compared to TN-IDC.

## Introduction

Triple-negative breast cancer (TNBC) accounts for 10 to 20% of all breast cancer cases [1, 2] and generally exhibits aggressive clinical behavior with a 5-year overall survival of approximately 75% [3, 4]. TNBC includes heterogeneous subtypes of breast cancer, and several demographic and tumor characteristics influence outcomes [5]. While the majority of TNBC is ductal histology, a small subset of TNBC is noted to be of lobular subtype. At present, the clinical characteristics and outcomes of triple-negative invasive lobular carcinoma (TN-ILC) of the breast are not well-characterized.

Invasive lobular carcinoma (ILC) of the breast, which comprises 10-15% of all breast cancers [6,7], are generally low grade and hormone receptor-positive, with up to 95% expressing the estrogen receptor (ER) and up to 70% expressing the progesterone receptor (PR) [8-10]. This makes TN-ILC an uncommon entity, with prior studies suggesting that TN-ILC accounts for approximately 2% of all TNBCand 0.1% of all breast cancer cases [11, 12]. Due to the rarity of the tumor, there are no well-established standards for the treatment of TN-ILC. Thus management of TN-ILC is often extrapolated from the treatment of triple-negative invasive ductal carcinoma (TN-IDC) of the breast. The role of adjuvant or neoadjuvant chemotherapy has been questioned in ER-positive ILC [13, 14], but the role of chemotherapy in TN-ILC has not been specifically studied. Likewise, response to neoadjuvant chemotherapy has been shown to be poor in ER-positive ILC in prior studies [15, 16], but data remains scarce for TN-ILC. ILC typically has a low proliferation rate and distinct molecular biology compared to IDC [17], and therefore it can be postulated that patients with loco-regional TN-ILC may not derive the same level of benefit from chemotherapy observed in TN-IDC.

Hence, in this study, we aimed to compare the response to neoadjuvant chemotherapy and overall survival between TN-ILC and TN-IDC and identify prognostic factors in TN-ILC.

## Methods

### Patient selection:

The current study utilized the National Cancer Database (NCDB) to identify women between 18 and 99 years of age diagnosed with pathological TNM stage I to III TN-IDC or TN-ILC treated with mastectomy or breast-conserving surgery between 2010 and 2018. The NCDB is an oncology database supported by the American College of Surgeons which collects hospital-based registry data from Commission on Cancer accredited institutions [18]. Only histologically confirmed cases of ILC or IDC were included in the analysis based on the International Classification of Diseases codes 8520/3 and 8500/3, respectively, and all other histological subtypes of breast cancer including mixed invasive ductal lobular carcinoma were excluded. The details of the patient selection is presented in **Supplemental Figure 1**. TNBC classification in NCDB was based on ER and PR expression of less than 1% and HER2 negativity by immunohistochemistry or FISH [19]. Data on demographics, tumor characteristics, treatment, and survival were extracted from the NCDB. For patients who did not receive neoadjuvant chemotherapy, pathological TNM staging from the surgical specimen was utilized. For patients receiving neoadjuvant chemotherapy, clinical TNM stage at the time of diagnosis was utilized and assessment of response to neoadjuvant chemotherapy was based on Collaborative Stage Site-specific factor 21 [19]. The definitions of complete response, partial response, and no response as per the Site-specific factor 21 are provided in **Supplementary Methods**.

### Statistical analysis:

Descriptive statistics were used to describe the baseline characteristics of TN-ILC and TN-IDC. Fisher exact test was used to compare categorical variables, while the Mann-Whitney U test was used for continuous variables. The 3- and 5-year overall survival was assessed utilizing the Kaplan- Meier method and the overall survival was compared among groups using the log-rank test. To assess the prognostic factors for TN-ILC, univariate and multivariate Cox proportional hazards regression models were used to estimate hazard ratios (HR) and 95% confidence intervals (CI). The multivariate analysis included age at diagnosis, race, grade, TNM stage, and treatment modalities such as type of surgery, adjuvant chemotherapy, and radiation. For analysis of the TNBC cohort who received neoadjuvant chemotherapy, cross-tabulation was done between the histological type of breast cancer and responses to neoadjuvant therapy. Multivariate logistic regression analysis was performed to evaluate the factors associated with pathological complete response (pCR) to neoadjuvant chemotherapy in the TNBC cohort and TN-ILC cohort separately. Kaplan-Meier curves for survival were plotted for pCR, partial response, and no response after neoadjuvant chemotherapy and further compared based on the log-rank test. All analyses were performed using STATA.

## Results

### Patient Characteristics:

A total of 125,780 women with breast cancer fulfilled the eligibility criteria and were included in the final analysis with a median follow-up duration of 49.7 months. The demographic, clinical, and tumor characteristics of 1,671 patients of TN-ILC and 124,109 patients of TN-IDC are presented in Table 1. The median age at diagnosis of TN-IDC and TN-ILC was 58 and 67 years respectively (p<0.001). The proportion of black women within the TN-ILC cohort was lower compared to within the TN-IDC group (12% vs. 21%, p<0.001). Compared to TN-IDC, women with TN-ILC were less likely (p<0.05) to present with high-grade tumors and receive chemotherapy or radiation but were more likely to have a higher stage at presentation and undergo mastectomy (Table 1). Approximately one-third of patients with TN-ILC and 19% of patients with TN-IDC did not receive chemotherapy. Further analysis demonstrated that older age at diagnosis, well-differentiated tumors, stage I breast cancer, lobular histology, and omission of radiation were associated with lower odds of receiving chemotherapy in the entire dataset.

### Comparison of overall survival between TN-ILC and TN-IDC:

In univariate analysis, women with TN-ILC were noted to have worse OS compared to women with TN-IDC (3-year OS: 80.1% vs 86.4%; 5-year OS: 70.5% vs 79.2%, p<0.001, **Supplemental Figure 2**). However, in multivariate cox-proportional hazard regression analysis adjusting for age at diagnosis, race, grade, TNM stage, and treatment modalities such as type of surgery, chemotherapy, and radiation, a significant difference in OS was not observed between women with TN-ILC and TN-IDC (HR 0.96, 95% CI 0.88-1.05, p=0.44, **Supplemental Table 1**).

### Prognostic factors in TN-ILC:

**Figure 1:**
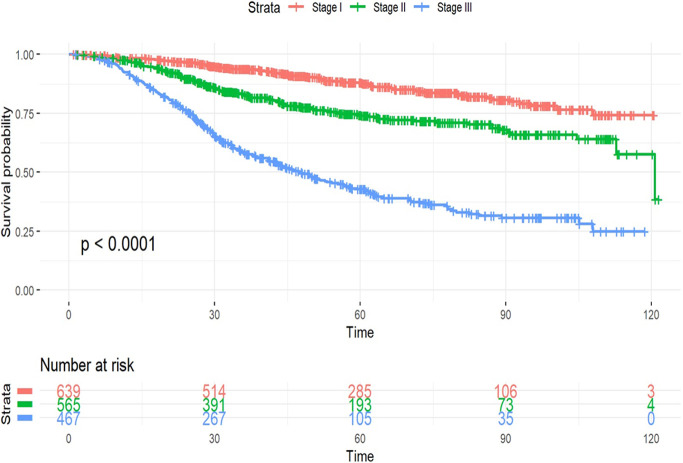
Overall survival by TNM-stage in women with TN-ILC

The 5-year OS among women with TN-ILC for pathological stages I, II and III was 88.2%, 74.3% and 42.8%, respectively (Figure 1). In multivariate analysis, black race (HR 1.51, 95% CI 1.16-1.95, p=0.002) and higher TNM stage at presentation (HR: 2.13, 95% CI 1.59-2.83, p<0.001 for Stage II and HR: 6.80, 95% CI 5.04-9.17, p<0.001 for Stage III, compared to Stage I) were associated with worse prognosis among women with TN-ILC, whereas receipt of chemotherapy (HR 0.63, 95% CI 0.49-0.79, p<0.001) and radiation (HR 0.79, 95% CI 0.64-0.99, p=0.04) were associated with better OS (Table 2). Stratified analysis by TNM stage was further performed to assess the prognostic impact of various covariates within each TNM stage in multivariate analysis. Administration of chemotherapy was associated with better OS in women with stage II (HR 0.48, 95% CI 0.31-0.73, p=0.001) and stage III (HR 0.60, 95% CI 0.42-0.87, p=0.007) TN-ILC, but the survival benefit with chemotherapy was not observed for women presenting with stage I TN-ILC (**Supplemental Table 2**). Radiation therapy was associated with better OS in stage III TN-ILC (HR 0.55, 95% CI 0.41-0.73, p<0.001) only (**Supplemental Table2**).

### Role of neoadjuvant chemotherapy in TN-ILC

**Figure 2:**
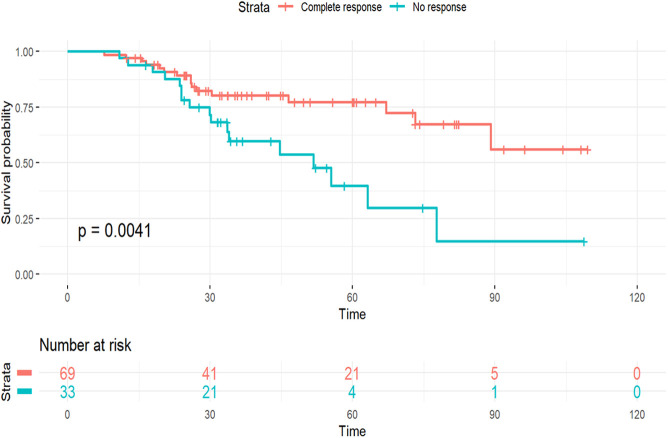
Overall survival by pathological response to neoadjuvant chemotherapy among women with triple-negative invasive lobular carcinoma of the breast

After the exclusion of women who did not receive neoadjuvant chemotherapy or for whom neoadjuvant chemotherapy status was unknown, a total of 298 women with TN-ILC and 27,754 with TN-IDC who received neoadjuvant chemotherapy were evaluated for pathological response to neoadjuvant chemotherapy (**Supplemental Table 3**). pCR was observed in 23.1% of women with TN-ILC compared to 36.2% of women with TN-IDC. In multivariate logistic regression analysis adjusting for age at diagnosis, race, grade, and clinical TNM stage of the tumor, women with TN-ILC were observed to have a lower odds of pCR (OR 0.53, 95% CI 0.34-0.82, p<0.001) with neoadjuvant chemotherapy compared to women with TN-IDC (**Supplemental Table 4**). In addition, lower odds of pCR were observed in women over the age of 50 compared to those aged 18-35 years (**Supplemental Table 4**). Among women with TN-ILC, the 5-year OS was 77.3% for women who achieved a pCR, and 39.8% for women without any response to neoadjuvant chemotherapy (Figure 2). TN-ILC patients with any residual disease, i.e. patients who did not achieve pCR, had significantly worse OS compared to patients who achieved pCR (5-year OS of 52.2% vs 77.3%, p=0.02).

## Discussion

In one of the largest studies on TN-ILC, we highlight important differences between TN-ILC and TN-IDC and demonstrate that the OS for TN-ILC is similar to TN-IDC after adjusting for demographic and tumor characteristics. Importantly, we found an association between chemotherapy administration and better OS in TN-ILC and identified that the odd of pCR following neoadjuvant chemotherapy is lower in TN-ILC compared to TN-IDC. These findings have significant implications for identifying appropriate treatment strategies for the management of TN-ILC.

Prior studies of women with TN-ILC have demonstrated similar [11, 12], better [20], and worse [21] OS compared to women with TN-IDC. The differences in the results of these studies could be related to the small sample size, selection biases and lack of adjustment for clinically relevant covariates. The present study is the largest to date comparing the outcomes of TN-ILC and TN-IDC while adjusting for several clinically relevant factors. While worse OS was noted for women with TN-ILC in univariate analysis, an OS difference was not observed in multivariate analysis suggesting that any difference in OS between the two groups is likely related to age at diagnosis and tumor stage at presentation.

This study highlights that a higher proportion of women with TN-ILC present with locally advanced disease compared to women with TN-IDC, which may be due to the low sensitivity of screening mammography for ILC compared to IDC [22, 23]. The higher rate of mastectomy among women with TN-ILC may also be related to the larger tumor size and the infiltrative nature of ILC observed on diagnostic imaging [24]. In addition, compared to women with TN-IDC, the median age at diagnosis of breast cancer was noted to be significantly older, while the proportion of black women was significantly lower among women with TN-ILC. These demographic differences in age and race suggest inherent differences in the environmental and genetic factors predisposing to TN-ILC and TN-IDC. Prior studies have identified a lower frequency of BRCA1 germline mutations and a higher frequency of CDH1 mutations among women with ILC compared to women with IDC [25, 26], but it is unclear if these findings apply to women with TN-ILC. A recent study on TN-ILC did show a high frequency of androgen receptor expression and ERBB2 gene mutations, with genetic alterations involving multiple genes in the ErbB signaling pathway [27]. A comprehensive analysis of the environmental and genetic factors, including the role of rare predisposition genes, is needed to understand the factors predisposing to TN-ILC.

This study supports the role of adjuvant or neoadjuvant chemotherapy among women with TN-ILC. While chemotherapy is considered the standard of care for the majority of patients with TNBC [11, 28, 29], its specific role in improving OS in TN-ILC was not previously defined. In particular, the benefit of adjuvant chemotherapy was primarily observed in women with stage II or III TN-ILC at presentation in this study. The lack of OS benefit noted in women with stage I TN-ILC may be due to the smaller effect size of chemotherapy in improving OS in this subset of women with generally favorable prognosis due to early diagnosis. The benefit of chemotherapy could still be present for women with larger tumors even within the stage I subset, similar to the observations from the treatment of women with TN-IDC [30]. The present study lacks the power required to evaluate the effect of chemotherapy on OS within these smaller subgroups. The improvement of OS with radiation in women with stage III TN-ILC is also consistent with prior reports from TN-IDC and TNBC [31, 32]. Similarly, the poorer outcome in black women with TN-ILC is also similar to findings from other studies of triple-negative breast cancer [33, 34], and could be related to socio-demographic factors or differences in tumor biology.

Over the past few years, neoadjuvant chemotherapy has become the standard of care in the treatment of women with triple-negative breast cancer as it helps in the assessment of prognosis [35] and adaption of adjuvant treatments [36-38] based on response to neoadjuvant treatment. In this study, we demonstrate that response to neoadjuvant chemotherapy correlates with long-term prognosis in women with TN-ILC, similar to prior findings [35]. However, interestingly, the possibility of achieving a pCR to neoadjuvant chemotherapy was significantly lower in women with TN-ILC compared to women with TN-IDC despite adjusting for several demographic and tumor variables. The finding of our study is comparable with the result of an NCDB analysis of women with all receptor subtypes of ILC, which showed a lower rate of pCR to neoadjuvant chemotherapy compared to IDC [39]. These findings suggest possible differences in tumor biology between TN-ILC and TN-IDC leading to differential responses to neoadjuvant chemotherapy. Prior studies have reported different, often limited, responses of ILC to chemotherapy and this has been explained based on the differences in their molecular profiling and genetic expression [5, 11]. The poor chemotherapy response has been attributed to the differential expression of androgen receptors and tumor-infiltrating lymphocytes [12, 40], but these findings need to be further investigated. It will be important to replicate this study in future TN-ILC patients who receive immune checkpoint inhibitors in combination with chemotherapy. In the meantime, the findings of this study suggest that the approach to chemotherapy, immune checkpoint inhibitors, and other targeted therapies should probably be similar between women with TN-ILC and TN-IDC until proven otherwise.

While a difference in pCR rates to neoadjuvant chemotherapy was observed between TN-ILC and TN-IDC, this did not translate into a difference in OS between the two groups. Since the rates of pCR were assessed in a small subset of patients receiving neoadjuvant chemotherapy whereas OS was evaluated in the entire dataset, it is possible that there may be a selection bias of relatively higher-risk patients with poor prognoses within the neoadjuvant group of TN-ILC compared to the neoadjuvant group of TN-IDC. In addition, while TN-ILC may be less chemosensitive compared to TN-IDC, it is possible that TN-ILC has a lower propensity for metastasis compared to TN-IDC which may have ultimately resulted in similar overall survival.

This study has a few limitations. Even though the overall study population was large, the sample size for the TN-ILC group, especially in the neoadjuvant category was small, which could result in a lack of power to detect differences for certain variables. The response to neoadjuvant chemotherapy was missing for several patients and standard residual cancer burden categories were not available. The lack of data on relapse is another important limitation, although OS could be considered a clinically meaningful outcome in patients with breast cancer especially considering the relatively poor prognosis of triple-negative breast cancer. In addition, genomics and other markers of tumor biology were not available for analysis in the NCDB. Furthermore, the triple-negative receptor status in this study was not centrally verified, and it is possible some of the cases were erroneously recorded as triple-negative, although the percentage of such errors is expected to be low.

## Conclusion

Women with TN-ILC have similar OS compared to women with TN-IDC after adjusting for tumor and demographic characteristics. While administration of neoadjuvant or adjuvant chemotherapy was associated with improved OS in TN-ILC, women with TN-ILC were less likely to achieve pCR to neoadjuvant compared to women with TN-IDC. These findings suggest that women with TN-ILC should be treated similarly to women with TN-IDC, but further studies evaluating the differences in tumor biology and responsiveness to chemotherapy and immune checkpoint inhibitors between the two subtypes of TNBC are needed to understand specific differences in outcomes.

## Figures and Tables

**Table 1:** Baseline characteristics of women with TN-ILC and TN-IDC

	TN-ILC, N= 1,671 (%)	TN- IDC, N=124,109 (%)	P-Value
**Age at diagnosis:**			< 0.001
Mean ± SD	66.4 ± 12.4	58.5 ± 13.6	
Median	67	58	
Range	27-90	18-90	

**Race:**			<0.001
White	1,347 (80.6%)	91,494 (73.7%)	
Black	200 (11.9%)	26,465 (21.3%)	
Other	113 (6.8%)	5,214 (4.2%)	
Unknown	11 (0.7%)	936 (0.8%)	

**Grade:**			0.0001
Well-differentiated	162 (9.7%)	1,798 (1.5%)	
Moderately differentiated	929 (55.6%)	19,126 (15.5%)	
Poorly differentiated	391 (23.4%)	97,853 (78.8%)	
Unknown	189 (11.3%)	5,332 (4.2%)	

**Overall TNM Stage:**			<0.001
Stage I	639 (38.2%)	58,763 (47.4%)	
Stage II	565 (33.8%)	50,809 (40.9%)	
Stage III	467 (28%)	14,537 (11.7%)	

**Type of surgery:**			0.0001
Breast conserving surgery	644 (38.6%)	66,058 (53.4%)	
Mastectomy	1,026 (61.4%)	57,821 (46.6%)	

**Chemotherapy:**			<0.001
Chemotherapy not administered	550 (32.9%)	23,883 (19.2%)	
Chemotherapy administered	1,099 (65.8%)	98,893 (79.7%)	
Unknown	22 (1.3%)	1,333 (1.1%)	

**Radiation:**			0.009
No	695 (41.6%)	47,710 (38.4%)	
Yes	976 (58.4%)	76,339 (61.6%)	

TN-ILC: Triple-negative Invasive Lobular Carcinoma; TN-IDC: Triple-negative Invasive Ductal Carcinoma

**Table 2:** Prognostic factors for overall survival among women with TN-ILC in multivariate analysis

	Hazard ratio^a^ (95% CI)	P-Value
**Age at diagnosis:**	1.01 (0.99-1.01)	0.09

**Race:**		
White	Reference	
Black	1.51 (1.16-1.95)	0.002
Other	0.41 (0.23-0.75)	0.004
Unknown	0.90 (0.28-2.87)	0.82

**Grade:**		
Well-differentiated	Reference	
Moderately differentiated	1.08 (0.74-1.58)	0.68
Poorly differentiated	1.47 (0.99-2.20)	0.05
Unknown	1.59 (1.03-2.46)	0.03

**Overall TNM Stage:**		
Stage I	Reference	
Stage II	2.13 (1.59-2.83)	<0.001
Stage III	6.80 (5.04-9.17)	<0.001

**Type of surgery:**		
Breast-conserving surgery	Reference	
Mastectomy	1.19 (0.93-1.53)	0.14

**Chemotherapy:**		
Not administered	Reference	
Administered	0.63 (0.49-0.79)	<0.001
Unknown	0.85 (0.40 – 1.75)	0.65

**Radiation:**		
No	Reference	
Yes	0.79 (0.64-0.99)	0.04

a : Hazard ratios for death are adjusted for all variables listed in the table in multivariate cox-proportional hazard regression analysis; CI: Confidence Interval

## Data Availability

The datasets generated during and/or analyzed during the current study are publicly available through the National Cancer Database.
